# Fentanyl-induced reward seeking is sex and dose dependent and is prevented by D-cysteine ethylester

**DOI:** 10.3389/fphar.2023.1241578

**Published:** 2023-09-19

**Authors:** Zackery T. Knauss, Caden J. Hearn, Nathan C. Hendryx, Fanan S. Aboalrob, Yazmin Mueller-Figueroa, Derek S. Damron, Stephen J. Lewis, Devin Mueller

**Affiliations:** ^1^ Department of Biological Sciences, Kent State University, Kent, OH, United States; ^2^ Department of Pediatrics, Division of Pulmonology, Allergy, and Immunology, Case Western Reserve University, Cleveland, OH, United States

**Keywords:** opioid use disorder (OUD), D-cysteine ethylester (D-CYSee), fentanyl, place conditioning, dose response, sex differences, substance abuse and addiction, conditioned place preference (CPP)

## Abstract

**Introduction:** Despite their inclination to induce tolerance, addictive states, and respiratory depression, synthetic opioids are among the most effective clinically administered drugs to treat severe acute/chronic pain and induce surgical anesthesia. Current medical interventions for opioid-induced respiratory depression (OIRD), wooden chest syndrome, and opioid use disorder (OUD) show limited efficacy and are marked by low success in the face of highly potent synthetic opioids such as fentanyl. D-Cysteine ethylester (D-CYSee) prevents OIRD and post-treatment withdrawal in male/female rats and mice with minimal effect on analgesic status. However, the potential aversive or rewarding effects of D-CYSee have yet to be fully characterized and its efficacy could be compromised by interactions with opioid-reward pathology.

**Methods:** Using a model of fentanyl-induced conditioned place preference (CPP), this study evaluated 1) the dose and sex dependent effects of fentanyl to induce rewarding states, and 2) the extent to which D-CYSee alters affective state and the acquisition of fentanyl-induced seeking behaviors.

**Results:** Fentanyl reward-related effects were found to be dose and sex dependent. Male rats exhibited a range-bound dose response centered at 5 µg/kg. Female rats exhibited a CPP only at 50 µg/kg. This dose was effective in 25% of females with the remaining 75% showing no significant CPP at any dose. Pretreatment with 100 mg/kg, but not 10 mg/kg, D-CYSee prevented acquisition of fentanyl seeking in males while both doses were effective at preventing acquisition in females.

**Discussion:** These findings suggest that D-CYSee is an effective co-treatment with prescribed opioids to reduce the development of OUD.

## Introduction

Synthetic opioids, such as fentanyl and fentanyl analogs (F/FA), are clinically administered to alleviate severe chronic and acute pain, and induce anesthesia (Lee and Ho, 2013; Fujii et al., 2019; Strang et al., 2020). However, their clinical efficacy is complicated by their inclination to induce analgesic tolerance, pleasure, and addictive states (Lee and Ho, 2013). In 2021, opioid use disorder (OUD) and subsequent overdose resulted in an estimated 80,411 deaths in the US, incurring a medical cost of more than 1–1.5 trillion dollars (Serdarevic et al., 2017; Torralva and Janowsky, 2019; CDC, 2020; National Center on Health Statistics, 2021; NIDA, 2023). OUD is a chronically relapsing disorder that is characterized by compulsive drug-seeking behavior that can be enhanced by drug abstinence and is maintained by encounters with drug-associated cues which elicit strong cravings and promote drug-seeking behaviors (Mueller et al., 2002; Zanda et al., 2021; Zhang et al., 2022). A large body of literature shows that sex differences impose clinically relevant changes in the vulnerability to OUD with women being prescribed and escalating to higher doses of opioid faster with greater craving and withdrawal symptoms compared to their male counterparts (Lee and Ho, 2013; Manubay et al., 2015; Serdarevic et al., 2017; Huhn et al., 2019a; Huhn et al., 2019b; Pisanu et al., 2019; Torralva and Janowsky, 2019; CDC, 2020; National Center on Health Statistics, 2021; Wightman et al., 2021). Despite this, sex differences in relation to the experience of opioid reward remain ambiguous with contradictory findings in both human and animal studies (Lee and Ho, 2013; Huhn et al., 2019a; Wightman et al., 2021). Since 1999, deaths resulting from synthetic opioid-induced overdose have increased by 103.2-fold in men, representing 73% of all synthetic opioid deaths in 2020, compared to women who have experienced a smaller 46.2-fold increase (Serdarevic et al., 2017; Barbosa-Leiker et al., 2018; Torralva and Janowsky, 2019; CDC, 2020; National Center on Health Statistics, 2021; NIDA, 2023). This divergence in the vulnerability to OUD and rate of overdose have been attributed to socio-economic pressures and differences in pain manifestation, coping, drug pharmacokinetics, and sensitivity to aversive drug effects (Lee and Ho, 2013; Manubay et al., 2015; Pisanu et al., 2019). However, little attention has been given to sex-dependent effects of fentanyl on opioid-induced reward with current literature largely focused on outcomes related to opioid-induced analgesia and respiratory depression (Manubay et al., 2015; Huhn et al., 2019a).

In the U.S., overdose from drugs of abuse is now the leading cause of accidental death in adults aged 18–50, resulting in ∼1,000 emergency visits and ∼91 deaths per day with more than 67% involving F/FA (Serdarevic et al., 2017; Torralva and Janowsky, 2019; CDC, 2020; National Center on Health Statistics, 2021). Overdose from opioids results in opioid-induced respiratory depression (OIRD) and cardiac toxicity which culminate in respiratory and cardiovascular failure. Opioid-induced respiratory conditions are reversed by administration of competitive opioid receptor antagonists such as naloxone and naltrexone (Barbosa-Leiker et al., 2018; Torralva and Janowsky, 2019). However, these treatments terminate opioid-induced analgesia, precipitate severe withdrawal symptoms, and are not effective against wooden chest syndrome (WCS, respiratory muscle rigidity and laryngospasm), uniquely resulting from the use of F/FA (Barbosa-Leiker et al., 2018; Torralva and Janowsky, 2019). In fact, despite the increased clinical and public availability of naloxone over the last decade, there has been no significant reduction in F/FA-related deaths (Torralva et al., 2020). As of 2022, an estimated 3 million people in the US and 16 million worldwide suffer from OUD (Azadfard et al., 2022). Medication-assisted treatments for OUD (i.e., methadone, buprenorphine, and ER naltrexone), which operate through direct antagonistic or agonistic action on the mu-opioid receptor, prevent subsequent clinical use of opioids in analgesia or anesthesia (Huhn et al., 2019a). These reactive intervention-based strategies for OUD are not preventative and burden patients with self-admittance and retention of treatment which is marginally effective with only 11% of people receiving treatment and less than 20.7% of those sustaining abstinence for at least 5 years (Zhu et al., 2018; Davis et al., 2021; Volkow, 2022). Accordingly, there is an urgent unmet medical need to develop drugs which can 1) reverse OIRD/WCS resulting from highly potent synthetic opioids without compromising analgesic efficacy or inducing aversive withdrawal states, and 2) prevent the development of and/or aid in the arrest of OUD.

Preclinical investigations have demonstrated that the thioester drug, D-cysteine ethylester (D-CYSee), delivers rapid and long-lasting recovery of respiratory function from OIRD in male and female rats with minimal effect on analgesic efficacy (Getsy et al., 2022c). These findings strongly indicate that D-CYSee and related thioesters (Mendoza et al., 2013; Gaston et al., 2021; Getsy et al., 2022b; Getsy et al., 2022c) act through an opioid receptor-independent mechanism that is metabolically distinct from those engaged by L-thiol esters which, while effective at recovering respiratory drive, induce maladaptive adverse effects on upper airway function (Gaston et al., 2021; Getsy et al., 2022c; Getsy et al., 2022b). Despite the clear therapeutic potential for D-CYSee in the recovery of respiratory drive following OIRD, the fact that it alters OIRD and that it readily passes the blood brain barrier suggests that indirect interactions with opioid-reward related effects are possible. Further, the potential aversive or rewarding behavioral effects of D-CYSee in the absence of opioids have yet to be fully characterized. While several previous studies have evaluated dose effects on the rewarding characteristics of fentanyl, these evaluations were often limited to male subjects and/or to a narrow dose range (Mucha and Herz, 1985; Vitale et al., 2003; Gaulden et al., 2021). Systematic analysis of the sex and dose specific differences in the rewarding properties of fentanyl could greatly aid in the development of studies evaluating treatment effects on fentanyl use disorder. Thus, experiments in the present study aimed to evaluate 1) the dose and sex dependent effects of fentanyl to induce rewarding states, and 2) the extent to which D-CYSee alters affective state and the acquisition of fentanyl-induced reward seeking.

## Materials and methods

### Subjects

Adult male (*n* = 98) and female (*n* = 79) Long-Evans outbred rats (ENVIGO, Indianapolis, IN), weighing approximately 200 g and 175 g at the start of testing, respectively, were housed individually in clear acrylic cages (65 cm × 24 cm × 15 cm) with food (Prolab RMH 3000, 5P00) and water *ad libitum*. The animal colony was maintained at a constant temperature (22°C ± 2°C) and humidity (50% ± 5% relative humidity) with a 14/10-h light/dark cycle (lights on at 0800 h). Animals were handled and weighed daily at time of testing. All procedures were approved by the Institutional Animal Care and Use Committee at Kent State University in compliance with National Institutes of Health guidelines.

### Apparatus

Behavioral conditioning and testing were conducted in a three-chamber apparatus (Med Associates) consisting of two visually distinct larger ‘conditioning’ chambers (13″ × 9″ × 11.5″) separated by a smaller intermediary chamber (6″ × 7″ × 11.5″). One of the conditioning chambers contained wire mesh flooring with white walls whereas the other had steel rod flooring with black walls. The center chamber had PVC plastic floors and gray walls. All floors were raised 1.5″, with removable trays placed underneath. Removable partitions were used to isolate the rats within specific chambers during conditioning. During conditioned place preference (CPP) trials, partitions were removed to allow free access to the entire apparatus for 15 min. The center box was illuminated by an overhead light and infrared beams located at the bottom of the walls allowed for quantitative assessment of animal preference for each compartment. During all phases of the experiment, the testing room was kept in semidarkness illuminated only by red light (240 lx) and a white noise generator (≈50 dB) was used to mask outside noise.

### Drug formulations and treatment regimens

Fentanyl citrate salt (Millipore Sigma, Cat#: F3886) was dissolved in sterile 0.9% saline at a stock/working concentration of 50 μg/mL and was diluted to working concentrations of 0.1, 0.5, 1.0, 5.0, 10, and 50 μg/mL and administered subcutaneously (s.c.) immediately prior to introduction to the conditioning chamber. D-cysteine ethylester hydrochloride (D-CYSee, Chem Impex, Cat#: 29770) was dissolved 15 min prior to treatment in sterile 0.9% saline at a concentration of 10 or 100 mg/mL and administered by intraperitoneal (i.p.) injection 15 min prior to introduction to the conditioning chamber. pH was calibrated to 7.4 for all treatments just prior to injection.

### Conditioning and testing

Following a 5-day acclimation and handling period, rats underwent a baseline preference test consisting of 15 min of unrestricted access to the full apparatus to assess initial chamber bias. The time spent in each chamber was recorded and analyzed by automated software (MED-PC IV). To account for an observed initial chamber preference, a biased CPP design was implemented in which drug chamber-pairings were assigned against each rat’s initial chamber preference. Next, animals were assigned in a pseudorandom and counter-balanced manner to receive one of twelve treatment regimens in the drug-paired chamber. In six of the groups, rats were conditioned to associate one chamber, but not another, with fentanyl (0.1, 0.5, 1.0, 5.0, 10, or 50 μg/kg, s.c.). An additional five groups were pretreated with D-CYSee (10 mg/kg or 100 mg/kg, i.p.) or saline 15 min prior to fentanyl administration (5 μg/kg male and 50 μg/kg female, s.c.). A control group was conditioned to associate both chambers with saline (1 mL/kg, s.c.). A preliminary fentanyl dose response study determined that doses of 5 μg/kg and 50 μg/kg fentanyl were optimal to induce reward in males and females, respectively, for treatment groups receiving both fentanyl and D-CYSee. Forty-eight h after baseline testing, rats were conditioned during eight daily sessions, wherein drug treatment or saline was administered in an alternating manner (four fentanyl and four saline pairings). Following injection, rats were confined to the allocated compartment for 30 min to allow for complete onset and experience of drug action. Starting 48 h after the final conditioning session, rats underwent daily extinction trials. Identical to the preference-baseline test, these daily sessions consisted of a single 15-min test during which animals had full apparatus access. Extinction trials were repeated until extinction criterion was met or until five extinction trials were completed. Extinction criterion measures were assessed by single day *t*-test comparison of time spent in drug versus saline-paired chambers under each treatment group. A group was considered to have extinguished if preference was not significant for two consecutive days.

### Data analysis

**FIGURE 1:**
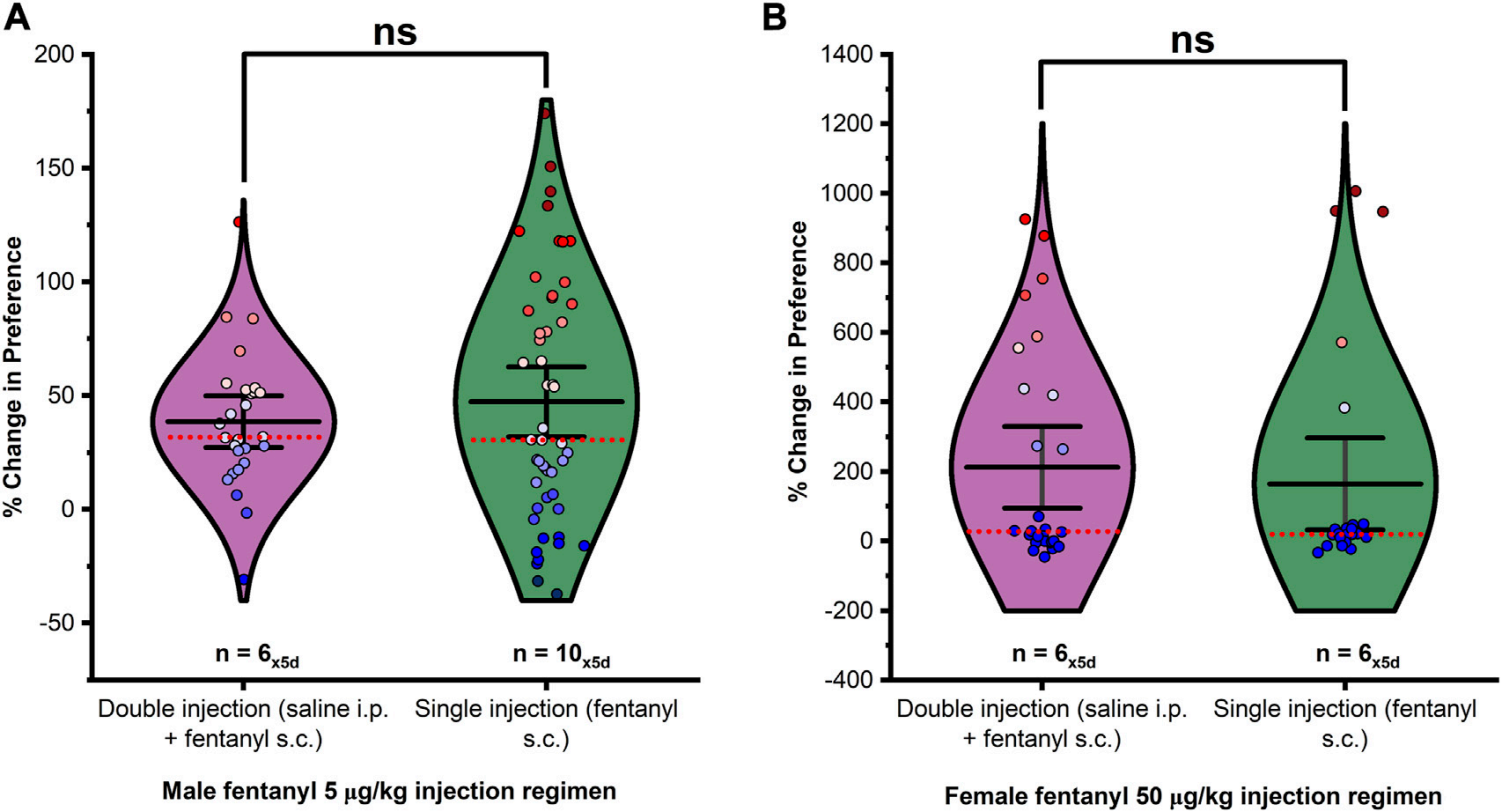
The presence or absence of a saline pretreatment injection had no significant effect on the percent change of preference in **(A)** males or **(B)** females. Data points represent the % change from each animal’s bias test during a single extinction trial, and data are presented as a summary of the 5 days of extinction testing. Black center line is the mean value of the normal distribution of the data, error bars in black are ±2*SE, and red dotted line is the median. *p < 0.05, **p < 0.01, ***p < 0.001.

To account for the third chamber variable omitted by analysis of time in drug-versus saline-paired chambers alone, we first calculated the decimal (DEC) percent of total time spent in the drug-paired chamber by each animal versus time spent overall in the apparatus during each testing session (See Eq. 1). Next, the percent relative change in preference from before conditioning for each animal under each extinction trial was calculated (See Eq. 2). No significant effect of injection on fentanyl conditioned place preference was observed in males (Figure 1A; F_1,76_ = 0.623, *p* > 0.05) or females (Figure 1B; F_1,51_ = 0.291, *p* > 0.05). Therefore, all data independent of injection was considered equivalent under matching drug treatment(s) and processed together. The effects of treatment on drug-seeking behavior were analyzed by an overall analysis of the percent relative change in preference over 5 days of extinction testing using a one-way ANOVA. Changes in preference between extinction days were assessed within each treatment by repeated measures ANOVA.

To reduce the risk of inflation of the F-value (type I error) due to unequal variances within the ANOVA, Mauchly’s test of sphericity was used to evaluate the assumption of sphericity, and violations were corrected for using Greenhouse-Geisser. All *post hoc* tests were conducted, when appropriate, using Fisher’s least significant difference (LSD) test.

$$\text{DEC}\%\text{ time in drug chamber}=\frac{\text{Drug}-\text{paired}\;\text{chamber}\;\text{time}\;\left(\sec\right)}{\text{Full}\;\text{experiment}\;\text{time}\;\left(\sec\right)}$$ (1)

$$\%\text{ Relative change in preference}=\left(\frac{\left(\text{Extinction}\;\text{trial}\;\text{Eq}1.-\text{Baseline}\;\text{pref}.\;\text{Eq}1.\right)}{\text{Baseline}\;\text{pref}.\;\text{Eq}1.}\right)x\;100\%$$ (2)

### Criteria for animal exclusion

A Grubbs extreme studentized deviant test (CL = 99.5) was used to evaluate data for outliers in each treatment group. Outliers were detected in the 10 μg/kg and 50 μg/kg fentanyl female treatment groups. The sole outlier within the 10 μg/kg fentanyl treatment group was likely the result of added stress due to an accidental home cage drop. Outliers within the 50 μg/kg fentanyl treatment group were more pervasive and were not isolated to any one set of animals and were only observed in females at this dose. Closer inspection of these data revealed a clean split centered at the mean between the female outliers and the bulk (75%) of remaining data. Further analysis of these data by test day showed that outliers were stable in preference over the full 5 days of testing. Thus, a mean split was used to separate the 50 μg/kg fentanyl female treatment group into two subgroups: 50 μg/kg fentanyl sensitive and 50 μg/kg fentanyl insensitive. As a result of apparatus, power, and/or computer failure a few data points were omitted or unrecoverable from each group (18 of 802 data points total). Within-group analysis revealed no significant change in preference between testing days resulting from any omitted or missing single trial data. One animal was removed in the female saline 1 mL/kg and fentanyl 50 μg/mL treatment group due to incorrect injection regimen.

## Results

### The effectiveness of fentanyl to induce reward seeking is sex and dose dependent

**FIGURE 2:**
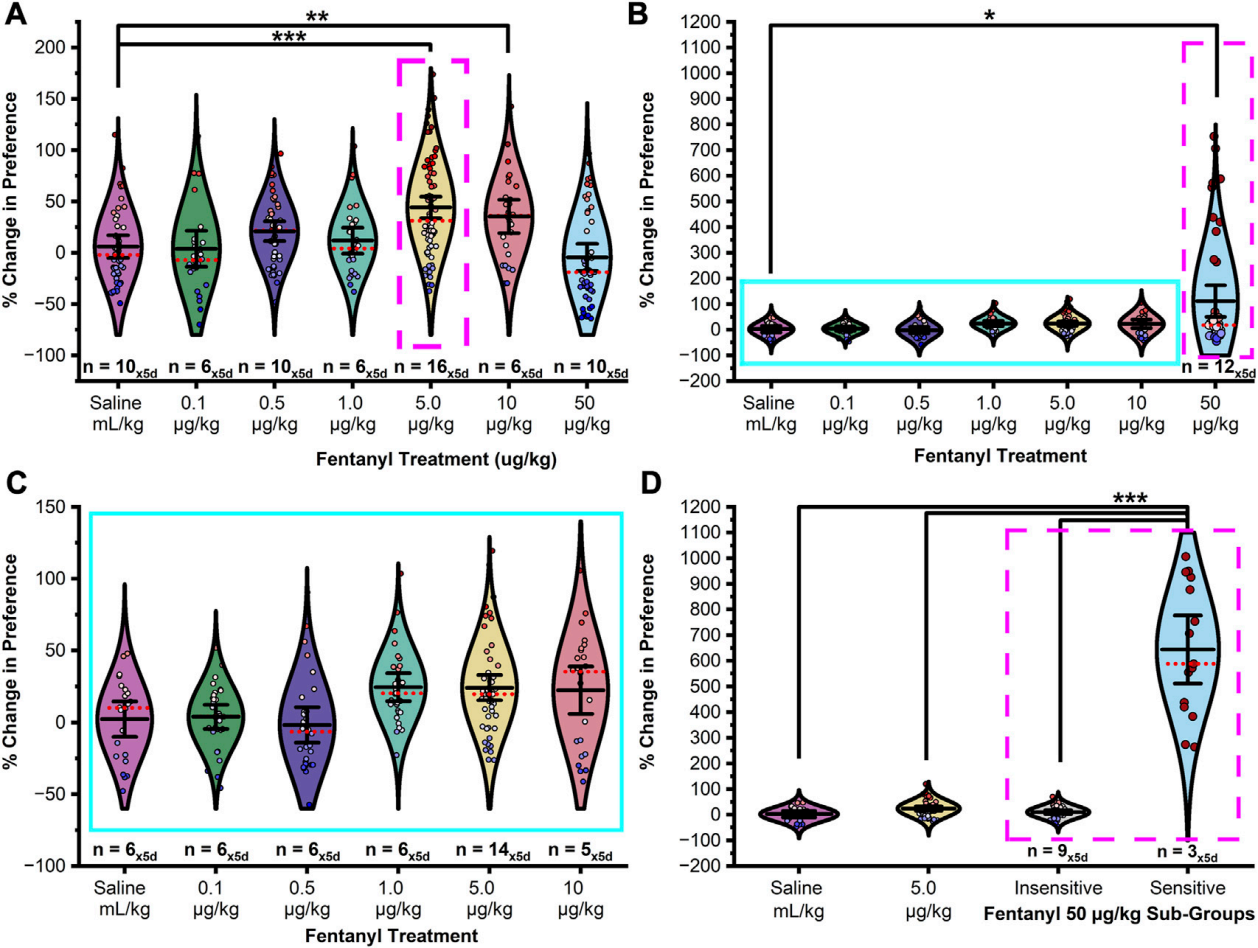
Fentanyl induces CPP in a sexually dimorphic and dose dependent manner. **(A)** In male rats, fentanyl doses of both 5 μg/kg and 10 μg/kg elicited a statistically significant change in preference compared to saline controls. Doses of 0.1 μg/kg, 0.5 μg/kg, 1 μg/kg, and 50 μg/kg did not induce a significant change from saline controls though a positive and negative trend was observed in preference on the left and right of 5 μg/kg fentanyl dose indicating that 5 μg/kg was the optimal dose in males to induce CPP (magenta box - broken line). **(B)** In female rats, the 50 μg/kg dose elicited a statistically significant change in preference from baseline-preference testing compared to saline controls. **(C)** Magnification of the blue box (solid line) in **(B)**. Whereas no dose tested within the range of 0.1–10 μg/kg elicited a significant response from saline controls a trend was observed in the 1–10 μg/kg treatment groups. **(D)** A mean split of the 50 μg/kg treatment group into fentanyl sensitive (25%) and insensitive (75%) subgroups revealed two statistically separate response phenotypes. Data points represent the % change from each animal’s bias test during a single extinction trial, and data are presented as a summary of the 5 days of extinction testing. Black center line is the mean value of the normal distribution of the data, error bars in black are ±2*SE, and red dotted line is the median. *p < 0.05, **p < 0.01, ***p < 0.001.

Following an initial baseline preference test, male and female rats were assigned to one of twelve treatment groups. In seven of the groups, rats were conditioned to associate one chamber, but not another, with fentanyl (0.1, 0.5, 1.0, 5.0, 10, and 50 μg/kg, s.c.). An additional five groups were pretreated with D-CYSee (10 mg/kg or 100 mg/kg, i.p.) or saline 15-min prior to fentanyl (5 μg/kg male and 50 μg/kg female, s.c.). A control group was conditioned to associate both chambers with saline (1 mL/kg, s.c.). Following conditioning, rats underwent five 30-min extinction sessions. To determine if fentanyl dose or sex had any effect on chamber preference, the percent relative change in preference from the baseline preference test and each extinction test was analyzed by ANOVA by treatment. As shown in Figure 2A, males showed a range-bound dose response suggesting a bell-shaped curve in that effective doses were restricted to a limited range centered at the 5 μg/kg dose with a significant difference in CPP between treatment levels (Figure 2A; F_10,412_ = 6.620, *p* < 0.0001). Fisher’s LSD confirmed the change in CPP resulting from 5 μg/kg fentanyl was significantly greater in magnitude than that of saline (*p* < 0.0001), or fentanyl at 0.1 μg/kg (*p* < 0.0001), 0.5 μg/kg (*p* = 0.002), 1 μg/kg (*p* = 0002), and 50 μg/kg (*p* < 0.0001). Treatment with 10 μg/kg fentanyl induced a significant CPP compared to saline controls (*p* = 0.002), and fentanyl at 0.1 μg/kg (*p* = 0.004), 1 μg/kg (*p* = 0.029), and 50 μg/kg (*p* < 0.0001). No significant difference in preference was found between 0.5 μg/kg fentanyl and the saline control group (*p* = 0.067), although a positive trend in preference was observed. No significant differences in preference were detected between any other groups (ps > 0.05).

In females, a mean split at the 50 μg/kg fentanyl dose (see Figures 2B–D) revealed two modes of drug responding (50 μg/kg sensitive and insensitive) with sensitive animals (∼25%) exhibiting a positive sigmoidal-like shaped curve with preference increasing with administered dose with a significant difference in preference between treatment groups (Figures 2B–D; F_11,351_ = 132.451, *p* < 0.0001). Post hoc tests confirmed that the change in CPP resulting from treatment with 50 μg/kg fentanyl (sensitive) was significantly greater in magnitude than that of saline treatment (*p* < 0.0001), or fentanyl at 0.1 μg/kg (*p* < 0.0001), 0.5 μg/kg (*p* < 0.0001), 1 μg/kg (*p* < 0.0001), 5 μg/kg (*p* < 0.0001), 10 μg/kg (*p* < 0.0001) or 50 μg/kg (insensitive; *p* < 0.0001). Surprisingly, no trend or significant differences in CPP were found between the saline control and 50 μg/kg fentanyl (insensitive) treatment group (*p* < 0.05). No significant differences were found in CPP from treatment with 1 μg/kg, 5 μg/kg, or 10 μg/kg fentanyl when compared to saline control (ps > 0.05). Further, no significant differences were found between any other groups (ps > 0.05).

### The stability of fentanyl reward seeking is prone to more variability early in extinction testing in both males and females

**FIGURE 3:**
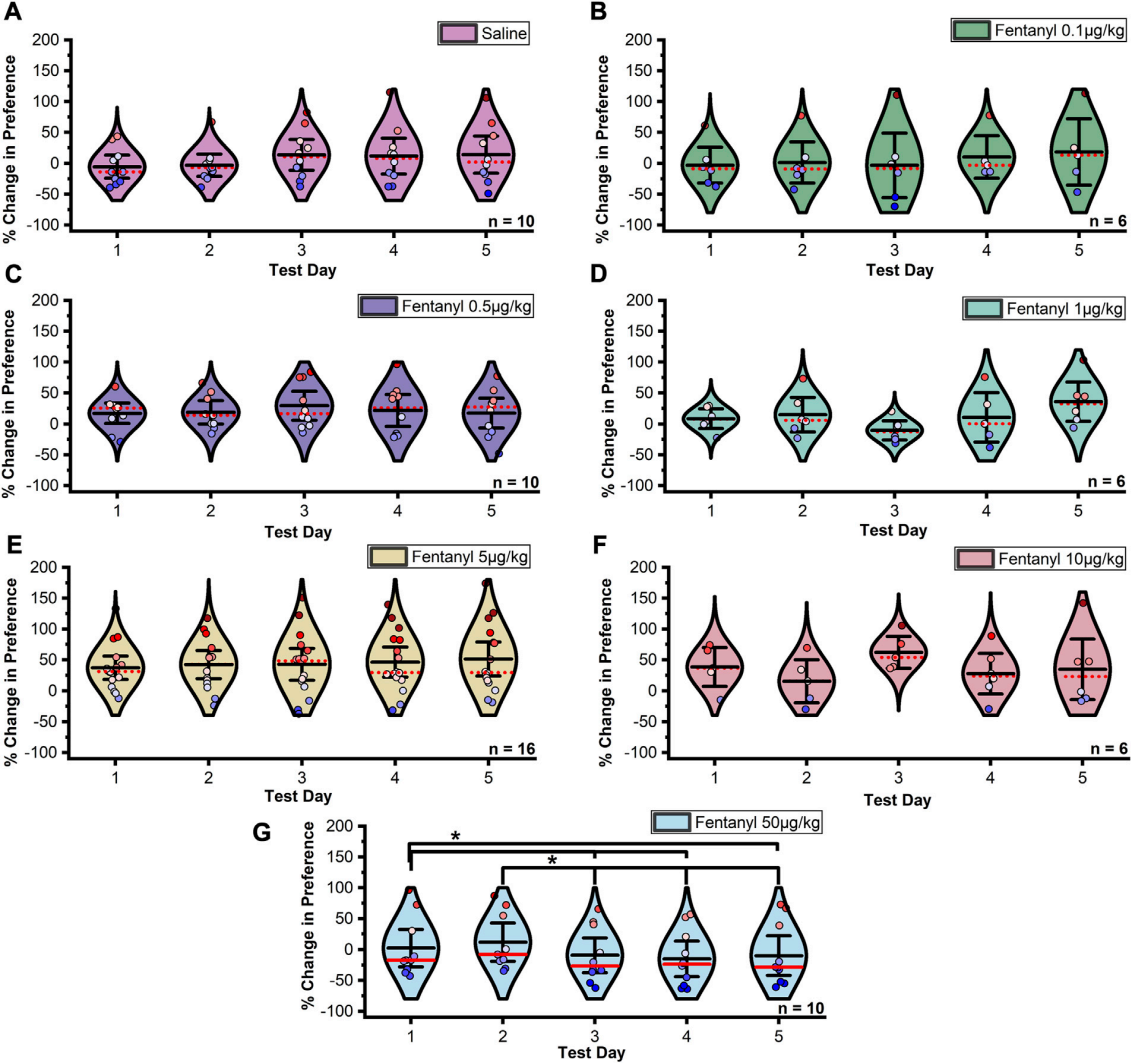
Data summarized in Figure 2A separated by individual extinction test. **(A–F)** No significant change in preference was found between days at doses of 0.1–10 µg/kg fentanyl or saline controls in male rats. **(G)** A significant downward shift and extinction in preference was detected between extinction days two and three under the 50 µg/kg fentanyl dose. This was not observed under either the 5 or 10 µg/kg dose resulting in a significant preference for the fentanyl paired chamber over saline controls. Black center line is the mean value of the normal distribution of the data, error bars in black are ±2*SE, and red dotted line is the median. *p < 0.05, **p < 0.01, ***p < 0.001.

The stability of CPP across extinction days within each treatment was assessed by repeated measures ANOVA. In males, Mauchly’s test indicated that the assumption of sphericity had been violated between extinction trials in the 5 μg/kg fentanyl (χ^2^_9_ = 34.072, *p* < 0.0001, ε = 0.505) and 10 μg/kg fentanyl (χ^2^_9_ = 18.541, *p* = 0.029, ε = 0.541) treatment groups, thus Greenhouse-Geisser corrected results for these groups are reported. The assumption of sphericity was not violated between extinction trials under any other group (ps > 0.05). A significant difference in the percent change in CPP between extinction trials was detected in the 50 μg/kg fentanyl treatment group (Figure 3; F_4,32_ = 7.397, *p* = 0.0002). Fisher’s LSD confirmed that the mean percent change in CPP significantly decreased in magnitude between extinction days one and three (*p* = 0.0235, one and four (*p* = 0.0027), one and five (*p* = 0.0359), two and three (*p* = 0.0006), two and four (*p* < 0.0001), and two and five (*p* = 0.001). No significant difference was observed between any other extinction test days (ps > 0.05).

**FIGURE 4:**
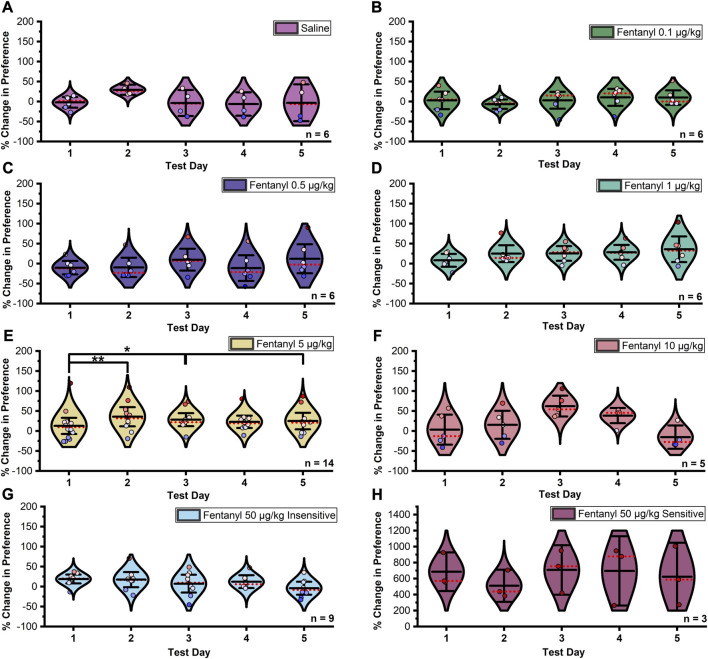
Data summarized in Figures 2B–D separated by individual extinction test. **(A–D)** and **(F–H)** No significant change in preference was found between days at doses of 0.1–5 µg/kg and 10–50 µg/kg fentanyl or saline controls in female rats. **(E)** A significant upward shift in preference was detected between extinction days one and two under the 5 µg/kg fentanyl dose. Although not significant in any other group, this trend was observed with the 0.5, 1, and 10 µg/kg fentanyl doses. Black center line is the mean value of the normal distribution of the data, error bars in black are ±2*SE, and red dotted line is the median. *p < 0.05, **p < 0.01, ***p < 0.001.

In females, Mauchly’s test indicated that the assumption of sphericity had been violated between extinction trials in the saline (χ^2^_9_ = 53.716, *p* < 0.0001, ε = 0.342), 0.5 μg/kg fentanyl (χ^2^_9_ = 17.866, *p* = 0.037, ε = 0.562), and 10 μg/kg fentanyl (χ^2^_9_ = 50.917, *p* < 0.0001, ε = 0.483) treatment groups, thus Greenhouse-Geisser corrected results for these groups are reported. The assumption of sphericity was not violated between extinction trials in any other group (ps > 0.05). Due to a limitation of the sample size the 50 μg/kg fentanyl sensitive group lacked sufficient degrees of freedom to complete a Mauchly’s test, thus sphericity was assumed. A significant difference in the percent change in preference between trials at the 5 μg/kg fentanyl dose (Figure 4; F_4,36_ = 2.771, *p* = 0.04) was observed. Post hoc tests confirmed that the mean percent change in preference significantly increased in magnitude between extinction days one and two (*p* = 0.003), one and three (*p* = 0.021), and one and five (*p* = 0.048). No significant effect was observed between extinction test days in any other group (ps > 0.05).

### D-CYSee prevents acquisition of fentanyl reward seeking in a sexually dimorphic and dose-dependent manner

**FIGURE 5:**
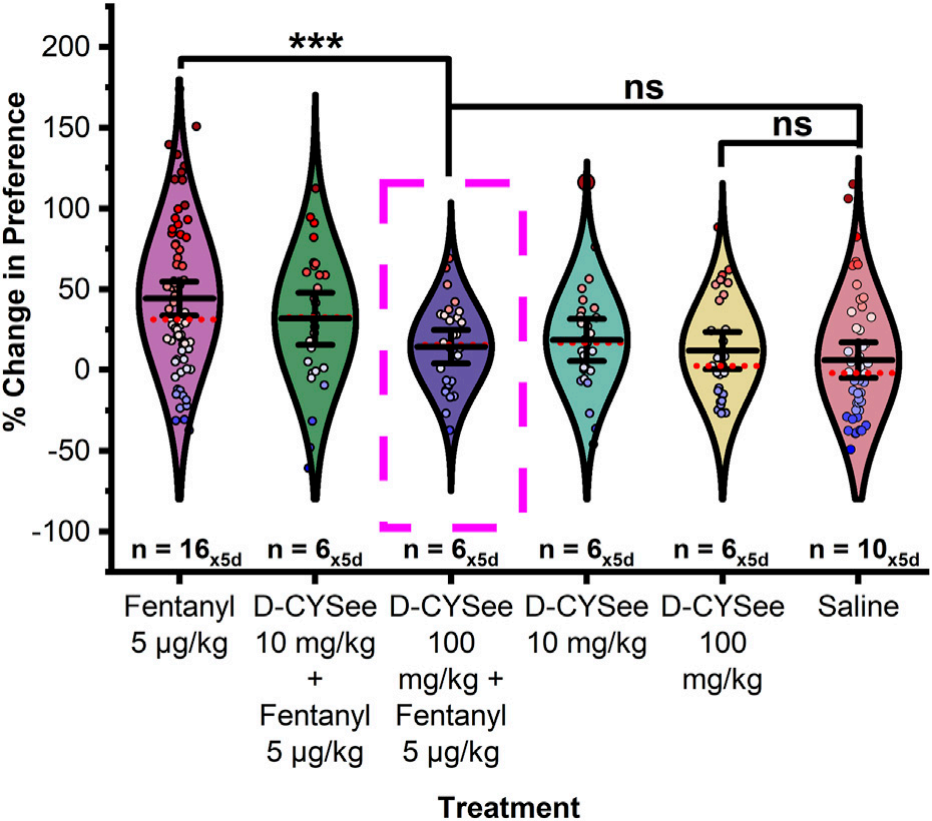
D-CYSee prevents acquisition of fentanyl-induced CPP in a dose dependent manner in male rats. Treatment with 100 mg/kg D-CYSee 15-min prior to fentanyl conditioning significantly decreased the preference for the fentanyl associated chamber compared to fentanyl controls. No significant difference was found in preference between saline controls, D-CYSee 100 mg/kg + fentanyl, and D-CYSee alone at 100 mg/kg. Treatment with 10 mg/kg D-CYSee 15-min prior to fentanyl conditioning did not significantly alter fentanyl preference though a trend of decreased preference for fentanyl was observed. Data points represent the % change from each animal’s bias test during a single extinction trial, and data are presented as a summary of the 5 days of extinction testing. Black center line is the mean value of the normal distribution of the data, error bars in black are ±2*SE, and red dotted line is the median. *p < 0.05, **p < 0.01, ***p < 0.001.

As shown in Figure 5, in males, there was a significant difference in the change in CPP between treatment groups (F_10,412_ = 6.620, *p* < 0.0001). Fisher’s LSD confirmed that male rats pretreated with 100 mg/kg D-CYSee (H-D-CYSee) 15-min prior to conditioning with 5 μg/kg fentanyl showed a significant decrease in preference compared to 5 μg/kg fentanyl controls (*p* < 0.0001). Critically, no significant difference was found between the H-D-CYSee+5 μg/kg fentanyl and saline (*p* = 0.378), H-D-CYSee+5 μg/kg fentanyl and H-D-CYSee (*p* = 0.818), or H-D-CYSee alone and saline (*p* = 0.523) treatment groups. Male rats pretreated with 10 mg/kg D-CYSee (L-D-CYSee) 15-min prior to conditioning with 5 μg/kg fentanyl did not show a significant change in CPP from 5 μg/kg fentanyl-treated controls (*p* = 0.159) although a negative trend in CPP was observed. A significant difference was observed between the saline control and the L-D-CYSee+5 μg/kg fentanyl treatment group (*p* = 0.007). No significant differences were detected between any other treatment groups (ps > 0.05).

**FIGURE 6:**
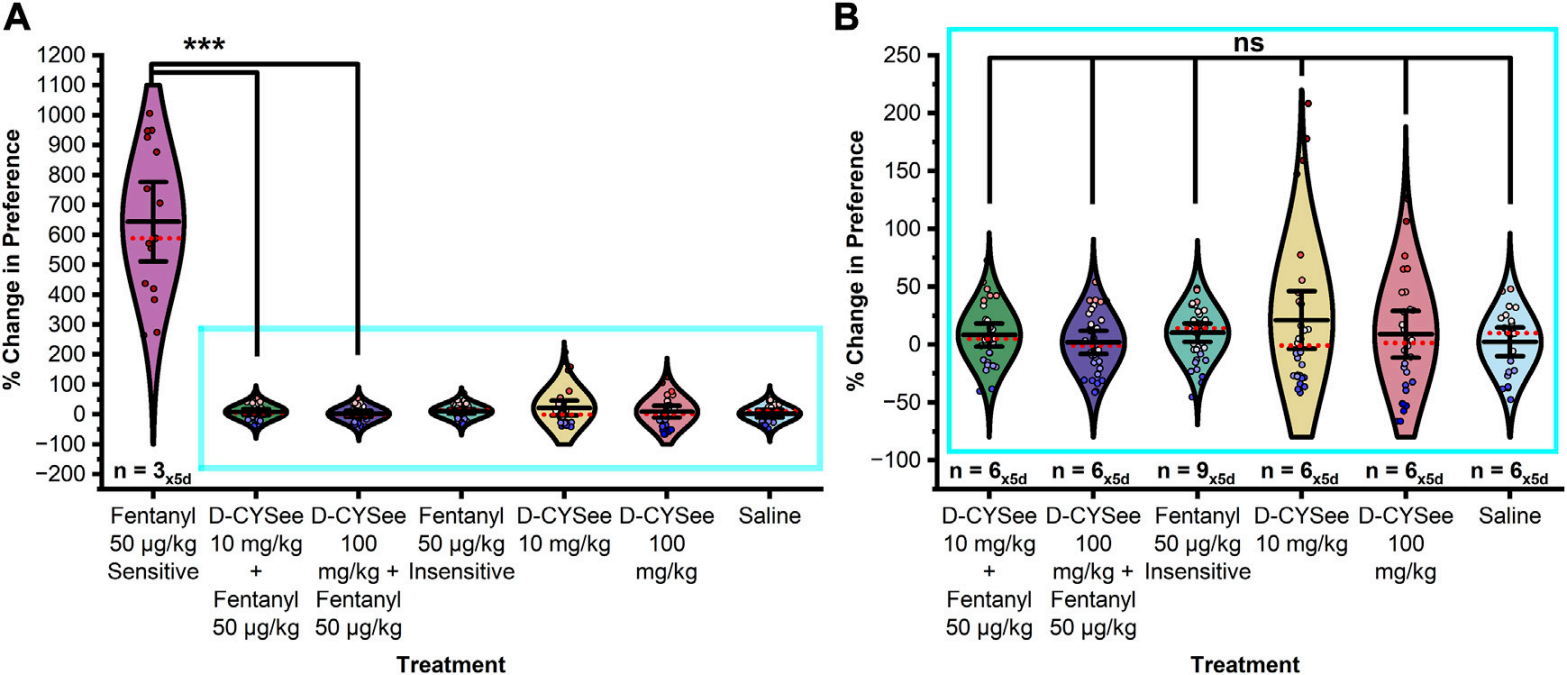
D-CYSee prevents acquisition of fentanyl-induced CPP in female rats. **(A)** Treatment with 100 mg/kg or 10 mg/kg D-CYSee 15-min prior to fentanyl conditioning significantly decreased the preference for the fentanyl associated chamber compared to fentanyl controls. No significant difference was found in preference between saline controls, D-CYSee 100 mg/kg + fentanyl, D-CYSee 10 mg/kg + fentanyl, D-CYSee alone at either 10 or 100 mg/kg, or 50 μg/kg fentanyl insensitive treatment groups. **(B)** Magnification of data without fentanyl sensitive females. Data points represent the % change from each animal’s bias test during a single extinction trial, and data are presented as a summary of the 5 days of extinction testing. Black center line is the mean value of the normal distribution of the data, error bars in black are ±2*SE, and red dotted line is the median. *p < 0.05, **p < 0.01, ***p < 0.001.

As shown in Figure 6, in females, a significant difference in CPP was detected between treatment groups (F_11,351_ = 132.451, *p* < 0.0001). Post hoc tests confirmed that females pretreated with either H-D-CYSee (*p* < 0.0001) or L-D-CYSee (*p* < 0.0001) 15-min prior to conditioning with 50 μg/kg fentanyl showed a significantly decreased CPP compared to fentanyl (sensitive) controls. No significant difference was detected between any other treatment group (ps > 0.05).

## Discussion

In the present study, we utilized a rat model of CPP to evaluate 1) the dose and sex dependent effects of fentanyl on reward-seeking behavior, and 2) the extent to which D-CYSee alters affective state and the acquisition of fentanyl-induced reward. Our results demonstrate significant differences in the fentanyl dose response between male and female Long-Evans rats. Fentanyl CPP in males followed a range-bound dose response centered at a dose of 5 μg/kg, with only doses of 5 μg/kg and 10 μg/kg eliciting a significant change in preference that also trended with doses of 0.5 μg/kg—1 μg/kg. Fentanyl doses above 10 μg/kg or below 0.5 μg/kg failed to induce any effect on preference in male rats. Conversely, fentanyl preference in female rats demonstrated a multifaceted dose response that was comprised of two sub-populations which responded in a divergent manner at higher fentanyl doses. While not significant, a linear trend toward preference was observed at doses from 1 μg/kg to 10 μg/kg. However, at a dose of 50 μg/kg a dual modality was observed wherein 25% of animals showed a significant CPP with the remaining 75% showing no difference in preference from saline control animals. No consistent changes or trends in preference were observed across the testing period of 5 days in males or females under any treatment condition, although test day one showed greater variability than later test days. Pretreatment with D-CYSee was effective at preventing the acquisition of fentanyl seeking in both male and female animals, and in the absence of fentanyl, D-CYSee induced no change in affective state. Critically, in males, but not females, D-CYSee exhibited a dose dependency wherein 100 mg/kg but not 10 mg/kg prevented the acquisition of fentanyl CPP. A partial non-significant reduction in preference compared to fentanyl controls was observed under the 10 mg/kg dose, whereas 100 mg/kg D-CYSee prevented fentanyl CPP. Critically, a dose dependency for D-CYSee was not observed in females as both the low and high dose were equally effective at preventing the acquisition of fentanyl CPP. These data suggest that males are more at risk for the development of OUD from lower dose fentanyl than females, and that females exhibit a dual modality in opioid reward experience. That is, a greater percentage of females are resistant to the rewarding effects of fentanyl at higher doses while a smaller percentage find these doses tremendously rewarding.

**FIGURE 7:**
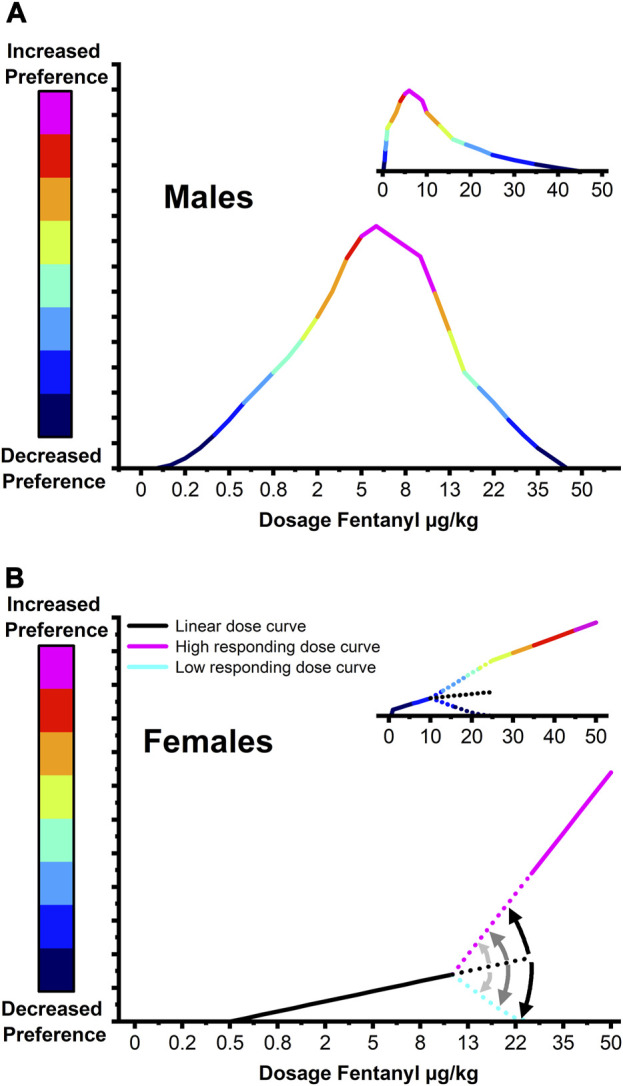
Proposed model of fentanyl reward dose response curves in male and female rats. **(A)** In males, fentanyl preference follows a bell-shaped dose-response curve centered at a dose of 4–6 μg/kg. **(B)** Females exhibit a transitional fentanyl dose response curve in which low doses of fentanyl (≈1 μg/kg–10 μg/kg) induce a low-grade preference that scales linearly. Doses above 10 μg/kg provoke a divergent response wherein animals progressively fall away from the linear model splitting into high preference or low-zero preference fentanyl response groups. Large graphs depict a nominal scale model over tested fentanyl doses while small graphs depict the model with dose scaled linearly.

Over the last few decades, several studies have utilized the CPP paradigm to evaluate the impacts of mu-opioid receptor agonists such as fentanyl on contextual preference and reward (Finlay et al., 1988; Vitale et al., 2003; Rech et al., 2011; Gaulden et al., 2021). The earliest study assessed the motivational properties of fentanyl at doses of 0.25, 1, 4, and 16 μg/kg in male Sprague Dawley rats and found a nearly identical range-bound fentanyl dose response centered at 4 μg/kg (Figure 7A; Mucha and Herz, 1985). Studies since have assumed equivalence between the male and female dose response curves employing only a few doses of fentanyl between 0.25 μg/kg and 16 ug/kg (Finlay et al., 1988; Vitale et al., 2003; Rech et al., 2011; Gaulden et al., 2021). Recent work found that significant fentanyl CPP could be elicited in females with a dose of 16 μg/kg but not 4 μg/kg (Gaulden et al., 2021). Interestingly, the data at the 16 μg/kg but not 4 μg/kg dose showed greater variability in which 25% (*n* = 4) expressed a preference above the standard error of the mean (SEM), ≈31% (*n* = 5) expressed a preference below the SEM, and 37% (*n* = 7) exhibited increased preference within the SEM. Critically, the data centered around the mean closely aligns with our linear dose response at lower fentanyl doses while those above and below the standard error are similar to, with smaller effect size, our findings at 50 μg/kg. Taken together these findings support a model of a transitional fentanyl dose response curve in female rats (Figure 7B) that exhibit a low-grade linear relationship with induced CPP at low doses (≈1 µg/kg-10 μg/kg). Doses above 10 μg/kg provoke a divergent response wherein animals progressively fall away from the linear model splitting into high preference or low-zero preference fentanyl response groups. This dual modality in fentanyl responding is consistent with findings showing that women with OUD are more motivated to obtain opioids and that female rats will self-administer more fentanyl than males and express higher levels of drug seeking (Lee and Ho, 2013; Serdarevic et al., 2017; Huhn et al., 2019a; Huhn et al., 2019b; Townsend et al., 2019; Bakhti-Suroosh et al., 2021; Towers et al., 2021; Wightman et al., 2021; NIDA, 2022).

Current literature on differences between female-female and female-male reward experience to drugs of abuse including opioids have largely centered on estrous cycling. Supporting this, recent work showed that the estrous cycle phase during the first exposure to fentanyl was predictive of elicited preference (Gaulden et al., 2021). However, the response pattern previously discussed within their 16 μg/kg treatment group was maintained within both estrus and non-estrus groups. Thus, while the estrous cycling may impact the magnitude of the elicited preference at lower doses, it does not appear to underlie the divergent responding seen in females in response to high-dose fentanyl. Recent findings have identified sex differences in the behavioral inhibition system centered in the prefrontal cortex, and in hippocampal plasticity and cognitive processing which are critical for opioid-reward processing and the formation of a CPP (Giacchino and Henriksen, 1998; Euston et al., 2012; Gonzalez et al., 2014; Baldo, 2016; Otis et al., 2018; Nam et al., 2019; Yagi and Galea, 2019; Jung et al., 2022). These functional and morphological discrepancies between males and females in these regions may underlie the observed sex differences in opioid-reward processing and the efficacy of D-CYSee (Yagi and Galea, 2019; Jung et al., 2022). Further evaluation of the impacts of these sex differences on reward processing are needed to better inform the clinical application of opioids and the development of treatment(s) for OUD, OIRD and WCS. Further, the successful translation of these models to humans could greatly inform the clinical application of opioids in pain management and clarify the divergence between the predicted vulnerability to OUD, and the number of women who advance to OUD and overdose.

Current non-preventative treatment strategies for OUD and OIRD are less effective in females (Barbosa-Leiker et al., 2018; Huhn et al., 2019a; Hoopsick et al., 2020; Davis et al., 2021). Our data demonstrate that D-CYSee, while effective at preventing fentanyl reward in both sexes, is more effective in female rats. D-CYSee prevents the acquisition of fentanyl-induced CPP in both males and females suggesting that D-CYSee could be an effective co-treatment with prescribed opioids to reduce the development of OUD. D-CYSee has been shown to effectively and persistently reverse OIRD in rat and mouse models (Getsy et al., 2022c). As a cysteine derivative, D-CYSee shares properties with N-acetyl-L-cysteine (NAC) which has also been shown to reduce OIRD (Getsy et al., 2022a) and cocaine-seeking behavior (Madayag et al., 2007; Amen et al., 2011; Reichel et al., 2011; Schmaal et al., 2012; Corbit et al., 2014; Reissner et al., 2015). Thus, D-CYSee may act in a similar fashion to NAC through normalization of glutamate and restoration of glutamate uptake by the glutamate transporter 1 (GLT-1) (Schmaal et al., 2012; Reissner et al., 2015). Although limited data are available on the effects of NAC on opioid seeking, NAC has been shown to reduce reinstatement of heroin self-administration (Zhou and Kalivas, 2008). D-CYSee may share these same mechanisms, and the ethylester addition likely facilitates its effects through its cell/blood brain barrier permeability (Mendoza et al., 2013; Gaston et al., 2021; Jenkins et al., 2021; Getsy et al., 2022c). Thus, D-CYSee may be a more effective cysteine derivative than NAC at preventing OUD in addition to reversing OIRD in both males and females (Getsy et al., 2022c). Determining the molecular mechanism(s) by which D-CYSee affords its promising outcomes and whether it could be effective in the treatment of OUD will be the subject of future studies.

## Data Availability

The original contributions presented in the study are included in the article/Supplementary Material and further inquiries can be directed to the corresponding author.

## Abbreviations

CPP, conditioned place preference; D-CYSee, D-cysteine ethylester; DEC, decimal; F/FA, fentanyl and fentanyl analogs; H-D-CYSee, 100 mg/kg D-CYSee; i.p., intraperitoneal; L-D-CYSee, 10 mg/kg D-CYSee; LSD, least significant difference; NAC, N-acetyl-L-cysteine; OIRD, opioid induced respiratory depression; OUD, opioid use disorder; s.c, subcutaneous; SEM, standard error of the mean; WCS, wooden chest syndrome.

## References

[B1] Amen S. L. Piacentine L. B. Ahmad M. E. Li S. J. Mantsch J. R. Risinger R. C. (2011). Repeated N-acetyl cysteine reduces cocaine seeking in rodents and craving in cocaine-dependent humans. Neuropsychopharmacology 36, 871–878. 10.1038/npp.2010.226 21160464 PMC3052624

[B2] Azadfard M. Huecker M. R. Leaming J. M. (2023). Opioid addiction. Avaliable At: https://www.ncbi.nlm.nih.gov/books/NBK448203/ . 28846246

[B3] Bakhti-Suroosh A. Towers E. B. Lynch W. J. (2021). A buprenorphine-validated rat model of opioid use disorder optimized to study sex differences in vulnerability to relapse. Psychopharmacol. Berl. 238, 1029–1046. 10.1007/s00213-020-05750-2 PMC7786148 33404740

[B4] Baldo B. A. (2016). Prefrontal cortical opioids and dysregulated motivation: A network hypothesis. Trends Neurosci. 39, 366–377. 10.1016/j.tins.2016.03.004 27233653 PMC5818385

[B5] Barbosa-Leiker C. McPherson S. Layton M. E. Burduli E. Roll J. M. Ling W. (2018). Sex differences in opioid use and medical issues during buprenorphine/naloxone treatment. Am. J. Drug Alcohol Abuse 44, 488–496. 10.1080/00952990.2018.1458234 29672167 PMC6186400

[B6] CDC (2020). Overdose deaths accelerating during COVID-19. Avaliable At: https://www.cdc.gov/media/releases/2020/p1218-overdose-deaths-covid-19.html .

[B7] Corbit L. H. Chieng B. C. Balleine B. W. (2014). Effects of repeated cocaine exposure on habit learning and reversal by N-acetylcysteine. Neuropsychopharmacology 39, 1893–1901. 10.1038/npp.2014.37 24531561 PMC4059898

[B8] Davis J. P. Eddie D. Prindle J. Dworkin E. R. Christie N. C. Saba S. (2021). Sex differences in factors predicting post-treatment opioid use. Addiction 116, 2116–2126. 10.1111/add.15396 33405314 PMC8254742

[B9] Euston D. R. Gruber A. J. McNaughton B. L. (2012). The role of medial prefrontal cortex in memory and decision making. Neuron 76, 1057–1070. 10.1016/j.neuron.2012.12.002 23259943 PMC3562704

[B10] Finlay J. M. Jakubovic A. Phillips A. G. Fibiger H. C. (1988). Fentanyl-induced conditional place preference: Lack of associated conditional neurochemical events. Psychopharmacol. Berl. 96, 534–540. 10.1007/BF02180036 3149777

[B11] Fujii K. Koshidaka Y. Adachi M. Takao K. (2019). Effects of chronic fentanyl administration on behavioral characteristics of mice. Neuropsychopharmacol. Rep. 39, 17–35. 10.1002/npr2.12040 30506634 PMC7292323

[B12] Gaston B. Baby S. M. May W. J. Young A. P. Grossfield A. Bates J. N. (2021). d-Cystine di(m)ethyl ester reverses the deleterious effects of morphine on ventilation and arterial blood gas chemistry while promoting antinociception. Sci. Rep. 11, 10038. 10.1038/s41598-021-89455-2 33976311 PMC8113454

[B13] Gaulden A. D. Burson N. Sadik N. Ghosh I. Khan S. J. Brummelte S. (2021). Effects of fentanyl on acute locomotor activity, behavioral sensitization, and contextual reward in female and male rats. Drug Alcohol Depend. 229, 109101. 10.1016/j.drugalcdep.2021.109101 34628096 PMC8671359

[B14] Getsy P. M. Baby S. M. May W. J. Lewis T. H. J. Bates J. N. Hsieh Y. H. (2022a). L-NAC reverses of the adverse effects of fentanyl infusion on ventilation and blood-gas chemistry. Biomed. Pharmacother. 153, 113277. 10.1016/j.biopha.2022.113277 35724513 PMC9458628

[B15] Getsy P. M. Baby S. M. May W. J. Young A. P. Gaston B. Hodges M. R. (2022b). D-cysteine ethyl ester reverses the deleterious effects of morphine on breathing and arterial blood–gas chemistry in freely-moving rats. Front. Pharmacol. 13, 883329. 10.3389/fphar.2022.883329 35814208 PMC9260251

[B16] Getsy P. M. Young A. P. Grossfield A. Seckler J. M. Wilson C. G. Gaston B. (2022c). D-cysteine ethyl ester and D-cystine dimethyl ester reverse the deleterious effects of morphine on arterial blood-gas chemistry and Alveolar-arterial gradient in anesthetized rats. Respir. Physiol. Neurobiol. 302, 103912. 10.1016/j.resp.2022.103912 35447347 PMC9588175

[B17] Giacchino J. L. Henriksen S. J. (1998). Opioid effects on activation of neurons in the medial prefrontal cortex. Prog. Neuropsychopharmacol. Biol. Psychiatry 22, 1157–1178. 10.1016/S0278-5846(98)00053-0 9829295

[B18] Gonzalez M. C. Kramar C. P. Tomaiuolo M. Katche C. Weisstaub N. Cammarota M. (2014). Medial prefrontal cortex dopamine controls the persistent storage of aversive memories. Front. Behav. Neurosci. 8, 408. 10.3389/fnbeh.2014.00408 25506318 PMC4246460

[B19] Hoopsick R. A. Homish G. G. Leonard K. E. (2020). Differences in opioid overdose mortality rates among middle-aged adults by race/ethnicity and sex, 1999-2018. Public Health Rep. 136, 192–200. 10.1177/0033354920968806 33211981 PMC8093836

[B20] Huhn A. S. Berry M. S. Dunn K. E. (2019a). Review: Sex-based differences in treatment outcomes for persons with opioid use disorder. Am. J. Addict. 28, 246–261. 10.1111/ajad.12921 31131505 PMC6591072

[B21] Huhn A. S. Tompkins D. A. Campbell C. M. Dunn K. E. (2019b). Individuals with chronic pain who misuse prescription opioids report sex-based differences in pain and opioid withdrawal. Pain Med. 20, 1942–1947. 10.1093/pm/pny295 30690594 PMC6784741

[B22] Jenkins M. W. Khalid F. Baby S. M. May W. J. Young A. P. Bates J. N. (2021). Glutathione ethyl ester reverses the deleterious effects of fentanyl on ventilation and arterial blood-gas chemistry while prolonging fentanyl-induced analgesia. Sci. Rep. 11, 6985. 10.1038/s41598-021-86458-x 33772077 PMC7997982

[B23] Jung W. H. Lee T. Y. Kim M. Lee J. Oh S. Lho S. K. (2022). Sex differences in the behavioral inhibition system and ventromedial prefrontal cortex connectivity. Soc. Cogn. Affect Neurosci. 17, 571–578. 10.1093/scan/nsab118 34718814 PMC9164205

[B24] Lee C. W. Ho I. K. (2013). Sex differences in opioid analgesia and addiction: Interactions among opioid receptors and estrogen receptors. Mol. Pain 9, 45–10. 10.1186/1744-8069-9-45 24010861 PMC3844594

[B25] Madayag A. Lobner D. Kau K. S. Mantsch J. R. Abdulhameed O. Hearing M. (2007). Repeated N-acetylcysteine administration alters plasticity-dependent effects of cocaine. J. Neurosci. 27, 13968–13976. 10.1523/JNEUROSCI.2808-07.2007 18094234 PMC2996827

[B26] Manubay J. Davidson J. Vosburg S. Jones J. Comer S. Sullivan M. (2015). Sex differences among opioid-abusing patients with chronic pain in a clinical trial. J. Addict. Med. 9, 46–52. 10.1097/ADM.0000000000000086 25325300 PMC4310755

[B27] Mendoza J. Passafaro R. Baby S. Young A. P. Bates J. N. Gaston B. (2013). l-Cysteine ethyl ester reverses the deleterious effects of morphine on, arterial blood–gas chemistry in tracheotomized rats. Respir. Physiol. Neurobiol. 189, 136–143. 10.1016/J.RESP.2013.07.007 23892097 PMC4430552

[B28] Mucha R. F. Herz A. (1985). Motivational properties of kappa and mu opioid receptor agonists studied with place and taste preference conditioning. Psychopharmacol. Berl. 86, 274–280. 10.1007/BF00432213 2994144

[B29] Mueller D. Perdikaris D. Stewart J. (2002). Persistence and drug-induced reinstatement of a morphine-induced conditioned place preference. Behav. Brain Res. 136, 389–397. 10.1016/S0166-4328(02)00297-8 12429400

[B30] Nam M. H. Han K. S. Lee J. Won W. Koh W. Bae J. Y. (2019). Activation of astrocytic μ-opioid receptor causes conditioned place preference. Cell. Rep. 28, 1154–1166. 10.1016/j.celrep.2019.06.071 31365861

[B31] National Center on Health Statistics (2021). Drug overdose death rates - number of national drug overdose deaths involving select prescription and illicit drugs. Avaliable At: https://nida.nih.gov/research-topics/trends-statistics/overdose-death-rates#:∼:text=More%20than%20106%2C000%20persons%20in,illicit%20drugs%20and%20prescription%20opioids .

[B32] NIDA (2023). 2022-2026 NIDA strategic plan. Avaliable At: https://nida.nih.gov/download/48192/2022-2026-nida-strategic-plan.pdf?v=e805b277544c1d64161a8d95d03355b2 .

[B33] NIDA (2022). Sex and gender differences in substance use. Avaliable At: https://nida.nih.gov/publications/research-reports/substance-use-in-women/sex-gender-differences-in-substance-use .

[B34] Otis J. M. Fitzgerald M. K. Yousuf H. Burkard J. L. Drake M. Mueller D. (2018). Prefrontal neuronal excitability maintains cocaine-associated memory during retrieval. Front. Behav. Neurosci. 12, 119. 10.3389/FNBEH.2018.00119 29962941 PMC6010542

[B35] Pisanu C. Franconi F. Gessa G. L. Mameli S. Pisanu G. M. Campesi I. (2019). Sex differences in the response to opioids for pain relief: A systematic review and meta-analysis. Pharmacol. Res. 148, 104447. 10.1016/j.phrs.2019.104447 31499196 PMC12522423

[B36] Rech R. H. Briggs S. L. Mokler D. J. (2011). Fentanyl and spiradoline interactions in a place-conditioning black-white shuttle-box. Pharmaceuticals 4, 101–116. 10.3390/ph401101

[B37] Reichel C. M. Moussawi K. Do P. H. Kalivas P. W. See R. E. (2011). Chronic N-acetylcysteine during abstinence or extinction after cocaine self-administration produces enduring reductions in drug seeking. J. Pharmacol. Exp. Ther. 337, 487–493. 10.1124/JPET.111.179317 21303920 PMC3083102

[B38] Reissner K. J. Gipson C. D. Tran P. K. Knackstedt L. A. Scofield M. D. Kalivas P. W. (2015). Glutamate transporter GLT-1 mediates N-acetylcysteine inhibition of cocaine reinstatement. Addict. Biol. 20, 316–323. 10.1111/ADB.12127 24612076 PMC4437505

[B39] Schmaal L. Veltman D. J. Nederveen A. Van Den Brink W. Goudriaan A. E. (2012). N-Acetylcysteine normalizes glutamate levels in cocaine-dependent patients: A randomized crossover magnetic resonance spectroscopy study. Neuropsychopharmacology 37, 2143–2152. 10.1038/npp.2012.66 22549117 PMC3398721

[B40] Serdarevic M. Striley C. W. Cottler L. B. (2017). Sex differences in prescription opioid use. Curr. Opin. Psychiatry 30, 238–246. 10.1097/YCO.0000000000000337 28426545 PMC5675036

[B41] Strang J. Volkow N. D. Degenhardt L. Hickman M. Johnson K. Koob G. F. (2020). Opioid use disorder. Nat. Rev. Dis. Prim. 6, 3. 10.1038/s41572-019-0137-5 31919349

[B42] Torralva R. Eshleman A. J. Swanson T. L. Schmachtenberg J. L. Schutzer W. E. Bloom S. H. (2020). Fentanyl but not morphine interacts with nonopioid recombinant human neurotransmitter receptors and transporters. J. Pharmacol. Exp. Ther. 374, 376–391. 10.1124/jpet.120.265561 32513839 PMC7430447

[B43] Torralva R. Janowsky A. (2019). Noradrenergic mechanisms in fentanyl-mediated rapid death explain failure of naloxone in the opioid crisis. J. Pharmacol. Exp. Ther. 371, 453–475. 10.1124/jpet.119.258566 31492824 PMC6863461

[B44] Towers E. B. Bakhti-Suroosh A. Lynch W. J. (2021). Females develop features of an addiction-like phenotype sooner during withdrawal than males. Psychopharmacol. Berl. 238, 2213–2224. 10.1007/s00213-021-05846-3 PMC8295229 33907871

[B45] Townsend E. A. Negus S. S. Caine S. B. Thomsen M. Banks M. L. (2019). Sex differences in opioid reinforcement under a fentanyl vs. food choice procedure in rats. Neuropsychopharmacology 44, 2022–2029. 10.1038/s41386-019-0356-1 30818323 PMC6898628

[B46] Vitale M. A. Chen D. Kanarek R. B. (2003). Chronic access to a sucrose solution enhances the development of conditioned place preferences for fentanyl and amphetamine in male Long–Evans rats. Pharmacol. Biochem. Behav. 74, 529–539. 10.1016/S0091-3057(02)01034-1 12543216

[B47] Volkow N. (2022). Making addiction treatment more realistic and pragmatic: The perfect should not be the enemy of the good heath affairs. Avaliable At: https://nida.nih.gov/about-nida/noras-blog/2022/01/making-addiction-treatment-more-realistic-pragmatic-perfect-should-not-be-enemy-good .

[B48] Wightman R. S. Perrone J. Scagos R. Hallowell B. D. Krieger M. Li Y. (2021). Toxicological and pharmacologic sex differences in unintentional or undetermined opioid overdose death. Drug Alcohol Depend. 227, 108994. 10.1016/j.drugalcdep.2021.108994 34482038 PMC8464519

[B49] Yagi S. Galea L. A. M. (2019). Sex differences in hippocampal cognition and neurogenesis. Neuropsychopharmacology 44, 200–213. 10.1038/s41386-018-0208-4 30214058 PMC6235970

[B50] Zanda M. T. Floris G. Sillivan S. E. (2021). Drug-associated cues and drug dosage contribute to increased opioid seeking after abstinence. Sci. Rep. 11, 14825. 10.1038/s41598-021-94214-4 34290298 PMC8295307

[B51] Zhang J. J. Song C. G. Dai J. M. Li L. Yang X. M. Chen Z. N. (2022). Mechanism of opioid addiction and its intervention therapy: Focusing on the reward circuitry and mu-opioid receptor. MedComm (Beijing) 3, e148. 10.1002/mco2.148 PMC9218544 35774845

[B52] Zhou W. Kalivas P. W. (2008). N-acetylcysteine reduces extinction responding and induces enduring reductions in cue- and heroin-induced drug-seeking. Biol. Psychiatry 63, 338–340. 10.1016/j.biopsych.2007.06.008 17719565 PMC2709691

[B53] Zhu Y. Evans E. A. Mooney L. J. Saxon A. J. Kelleghan A. Yoo C. (2018). Correlates of long-term opioid abstinence after randomization to methadone versus buprenorphine/naloxone in a multi-site trial. J. Neuroimmune Pharmacol. 13, 488–497. 10.1007/s11481-018-9801-x 30094695 PMC6224303

